# Reversible tetraplegia after percutaneous nephrostolithotomy and septic shock: a case of critical illness polyneuropathy and myopathy with acute onset and complete recovery

**DOI:** 10.1186/1471-2369-14-36

**Published:** 2013-02-14

**Authors:** Hai Li, Li-Min Wu, Xiang-Bo Kong, Yi Hou, Rui Zhao, Hong-Yan Li, Hong-Liang Zhang

**Affiliations:** 1 Department of Urology, The China-Japan Union Hospital of Jilin University, Changchun, China; 2 Department of Neurology, The First Bethune Hospital of Jilin University, Jilin University, Xinmin Street 71#, 130021, Changchun, China

**Keywords:** Critical illness polyneuropathy, Critical illness myopathy, Percutaneous nephrostolithotomy, Sepsis, Guillain-Barré syndrome

## Abstract

**Background:**

Critical illness polyneuropathy (CIP) and critical illness myopathy (CIM) are complications causing weakness of respiratory and limb muscles in critically ill patients. As an important differential diagnosis of Guillain-Barré syndrome (GBS), CIP and CIM should be diagnosed with caution, after a complete clinical and laboratory examination. Although not uncommon in ICU, CIP and CIM as severe complications of percutaneous nephrostolithotomy (PNL) have not been documented in literature.

**Case presentation:**

A 48-year-old Chinese woman was referred to our hospital, complaining of occasional pain in the right lower back for one month. Lithiasis was diagnosed by ultrasonographical and radiological examinations on the urinary system. PNL was indicated and performed. The patient developed CIP and CIM on the fourth day after PNL. Early recognition and treatment of the severe complications contributed to a satisfactory recovery of the patient.

**Conclusion:**

This case expands our understanding of the complications of PNL and underscores the importance of differentiating CIP/CIM from GBS in case of such patients developing weakness after the treatment. Clinical characteristics and examination results should be carefully evaluated to make the diagnosis of CIP or CIM. Both anti-septic prophylaxis and control of hyperglycemia might be effective for the prevention of CIP or CIM; aggressive treatment on sepsis and multiple organ failure is considered to be the most effective measure to reduce the incidence of CIP/CIM.

## Background

Critical illness polyneuropathy (CIP) and critical illness myopathy (CIM) are frequent complications of critical illness involving both motor and sensory axons [1,2]. The cardinal clinical signs of CIP and CIM present as flaccid and symmetric weakness [2]. Reduction in or absence of deep tendon reflexes may sometimes herald the onset of limb weakness [3]. Epidemiological studies showed that CIP and/or CIM occur in approximately 70% of patients with sepsis or systemic inflammatory response syndrome (SIRS) [4], 60% of acute respiratory distress syndrome (ARDS) [5], and up to 100% of patients with multiple organ failure (MOF) [6]. CIP and CIM alone, or in combination prolong weaning from mechanical ventilation and physical rehabilitation [3].

Percutaneous nephrostolithotomy (PNL) is a procedure requiring indications of the treatment of large hard infected stones, obstruction-related stones, extracorporeal lithotripsy failures and stones related with anatomic variations [7]. Most of the complications of PNL are minor and without clinical repercussion [8]. Here, we report a case who developed CIP/CIM after PNL, which highlights the importance of understanding the complications of this procedure and identifying CIP/CIM in such patients developing weakness after treatment.

## Case report

**Figure 1:**
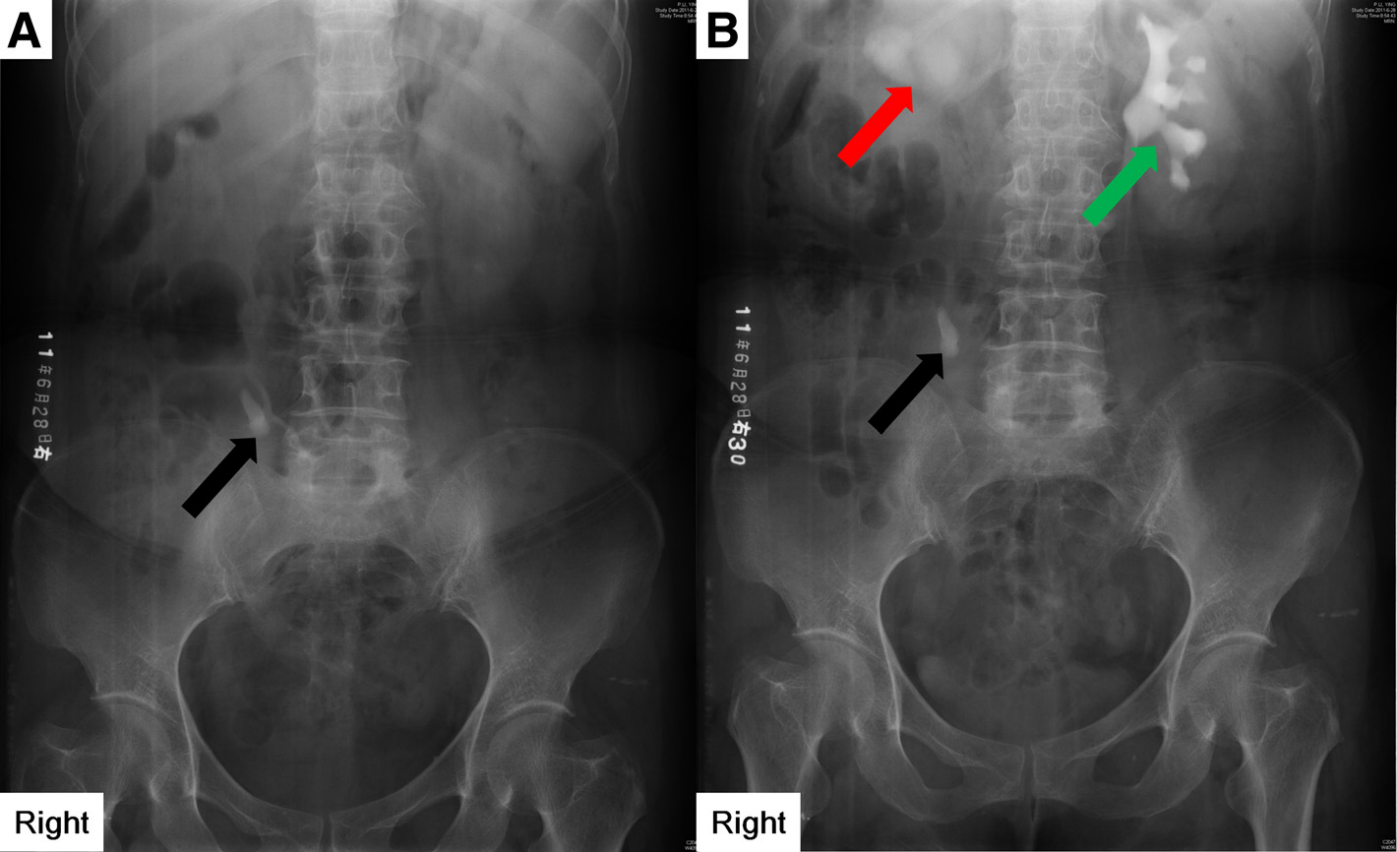
**Imaging manifestations of lithiasis.** Radiological evaluation was performed with IVU following a KUB. **A**. KUB before the intravenous injection of contrast. **B.** KUB 30s after the intravenous injection of contrast. A 2.5 cm × 1.0 cm high-density mass was shown in the right ureter at the L3 level (**A** and **B**, black arrows). Right renal pelvis was enlarged (**B**, red arrow). Lithiasis was diagnosed on the basis of the imaging features after excluding other diseases.

A 48-year-old Chinese woman was referred to our hospital, complaining of occasional pain in the right lower back for one month. The pain was blunt, lasting several minutes each time. The episodic pain occurred 5–6 times altogether. The patient was otherwise healthy, without any remarkable history of diseases. Ultrasonography of the urinary system revealed enlarged right kidney with dilated pelvis and ureter, and multiple calculus in the right kidney and ureter. Intravenous urography (IVU) following a KUB showed hydronephrosis and a 2.5 cm × 1.0 cm high-density mass in the right ureter at the L3 level (Figure 1). Lithiasis was diagnosed. PNL was indicated and performed. The whole procedure, lasting 40 minutes, was successful, with little blood loss.

Three hours after the surgery, the patient became feverish (body temperature up to 39.5 degrees centigrade). Emergent blood routine test was normal at that time, with blood samples simultaneously collected for hemoculture. The patient’s conditions deteriorated rapidly. Six hours after PNL, her blood pressure decreased to 80/60 mmHg, indicative of septic shock. The body temperature was as high as 41.0 degrees centigrade, and the heart rate was 110 beats per minute. An abnormal blood cytology was noted (WBC 13.97 × 10^9^/L, neurophil percentage 92.4%, lymphocyte percentage 4.6%, monocyte percentage 2.9%, hemoglobin concentration 10^5^ g/L, platelet 43 × 10^9^/L. Primaxin (imipenem and cilastatin, 0.5 g, q6h, i.v.d.) was administered to control the infection. On the 2^nd^ day after PNL, the patient’s conditions deteriorated even further; delirium, irritability and dyspnea appeared. The oxygen saturation decreased sharply to 60% and the patient fell comatose. Intubation and mechanical ventilation were immediately initiated. Five percent sodium bicarbonate (12.5 g) was intravenously administered to correct acidosis. The patient’s blood pressure could not be maintained above 90/60 mmHg by continuously infusing dopamine and metaraminol, use of methylprednisolone (40 mg, q.d., i.v.d., for 7 days) was initiated. On the 3^rd^ day after PNL, her blood WBC count was increased to 24.66 × 10^9^/L (neutrophil percentage 84.3%, lymphocyte percentage 6.7%, monocyte percentage 4.4%) and platelet count decreased to 32 × 10^9^/L. Level of creatinine in blood was elevated to 237.8 μmol/L. During the fourth day after PNL, absence of bilateral deep tendon reflexes was noted, whereupon neurologists were consulted. The Glasgow coma scale score was 7. She had even pupils with normal light reflex. Deep tendon reflexes were absent and pyramidal signs were negtive bilaterally; the Kennedy sign was positive. Since the patient was comatose, we could not examine the muscle strength of her extremities. ECG did not reveal any abnormalities. Laboratory tests showed an elevated serum level of aspartate transaminase (AST) (157 IU/L, normal range: 5–40 IU/L), creatine kinase (CK, 5452 IU/L, normal range: 25–200 IU/L), CK-MB (93.3 IU/L, normal range: 0–25 IU/L), and CK-MM (5393.3 IU/L, normal range: 0–150 IU/L ). Although CIP/CIM were suspected, Guillain-Barré syndrome (GBS) could not be excluded. IgG antibodies against GM1, GM1b, GD1a, and GalNAc-GD1a in serum, were negative. Considering the patient’s critical situation, electrophysiological investigations and lumbar puncture were not performed though advised. Intravenous immunoglobulin (0.4 g/(kg body weight)/day, i.v.d., for 5 days) was empirically administered. On the 7^th^ day after PNL, a methicillin resistant strain of *S. aureus.* was identified from her blood cultivation. Treatment combining vancomycin (1000 mg, b.i.d., i.v.d.) and fluconazole (50 mg, q.d., p.o.) was employed.

Two weeks after PNL, the patient turned conscious and partially cooperative to neurological examinations. The proximal motor power of her upper extremities was 1/5 and distal 2/5; the proximal motor power of her lower extremities was 1/5 and distal 2/5. Her muscle tone was reduced symmetrically in the limbs. Sensory tests including light touch, pain and vibration were not affirmatory. Lumbar puncture was performed, showing negative findings in the cerebrospinal fluid (CSF) with regard to cell count (WBC: 1 × 10^6^/L; RBC: 0 × 10^6^/L) and protein level (0.2 g/L). Since the patient was awake and cooperative, electrophysiological studies were performed. Decreased motor nerve action potential, reduced amplitude of the nerve conduction potentials (compound muscle action potential (CMAP)) and absent sensory nerve action potential (SNAP) with preserved conduction velocity were found, which pointed to a diagnosis of axonal motor polyneuropathy and myopathy. Combined with the elevated level of CK-MM in the serum, a diagnosis of CIP/CIM was thus established.

The patient’s blood pressure and body temperature returned to normal 18 days after PNL. Four weeks after PNL, she was weaned from mechanical ventilation when she was wide awake and could breathe spontaneously. Vancomycin and fluconazole were thereafter discontinued. Her muscle weakness gradually recovered. Six weeks after PNL, the patient still remained weak and unable to stand independently. Upon neurological examination, the proximal motor power of her upper extremities was 3/5 and distal 3/5; the proximal motor power of her lower extremities was 4/5 and distal 4/5. Her muscle tone was normal in the limbs. Bilateral deep tendon reflexes and pyramidal signs were absent. A second lumbar puncture was thus performed. The results were still normal. With an adjuvant therapy with vitamins 1 and 12, her muscle strength gradually recoverd in the following weeks and she could walk independently 2 months later.

## Discussion

We reported a case with acute-onset flaccid paralysis after PNL. We diagnosed our patient as CIP/CIM for the following reasons. First, sepsis and pulmonary dysfunction after PNL preceded symmetric weakness, indicating a role of MOF in the occurance of the clinical symptoms. Second, the patient manifested a rapid onset of muscle weakness, which differed from a typical progressive clinical course of GBS. Third, albumino-cytologic dissociation, an essential feature that contributes to a diagnosis of GBS, was not noted in CSF. Fourth, antibodies against GM1, GM1b, GD1a, and GalNAc-GD1a, which are associated with the axonal variants of GBS, were negative in serum. Fifth, the increased level of CK-MM and the absence of ECG positive findings, were strongly indicative of CIM. Finally, we diagnosed our patient as both CIP and CIM. Our diagnosis was based on electrophysiological findings as well as an elevated level of CK-MM in the serum.

PNL is a procedure that requires indications of the treatment of large, hard infected stones, obstruction-related stones, extracorporeal lithotripsy failures and stones related with anatomic variations [6]. Most of the complications of PNL are minor and without clinical repercussion [9]. Although fever and infection after PNL are common, progression to sepsis is rare. Sepsis may develop as a result of bacteriemia or endotoxemia after stone or urinary tract manipulation [10] and may trigger an inflammatory cascade leading to septic shock [11].

CIP is an acute axonal sensory-motor polyneuropathy and CIM is an acute primary myopathy with a continuum of myopathic findings, from myopathies with pure functional impairment and normal histology to myopathies with atrophy and necrosis [12]. According to Koch *et al.*, CIM was more frequent than CIP and most patients with CIP featured concomitant CIM [13]. Approximately 70-80% of critically ill patients develop CIP and a comparable percentage of them develop CIM [14]. Sepsis, SIRS, MOF and ARDS are the most common risk factors for CIP and/or CIM; prolonged ICU stay, medication and infections, are also major risk factors for CIP/CIM [15]. Especially, Gram-negative bacteremia is an independent risk factor for the development of CIP/CIM [16]. Other identified risk factors include female sex, hyperglycemia, severe illness, long duration of organ dysfunction, renal failure and renal replacement therapy, hyperosmolarity, malnutrition, low serum albumin, parenteral nutrition, vasopressor and catecholamine support, and septic encephalopathy, etc. [17,18]. Although excessive use of corticosteroids may lead to increased incidence of CIM, short-term use of methylprednisolone was meant to treat the septic shock in our case. Since the time interval between the initiation of methylprednisolone therapy and the onset of paralysis is quite short, we do not believe that the onset of CIM was due to methylprednisolone itself.

The pathogenesis of CIP and CIM is elusive, possibly involving microcirculatory changes, metabolic alterations, electrical abnormalities, and bioenergetic failure [19]. A definite diagnosis of CIP requires the following criteria fulfilled: a. The critically ill patient develop limb weakness or difficulty in weaning after non-neuromuscular causes such as heart and lung diseases have been excluded; b. EMG shows axonal motor and sensory polyneuropathy; c. A decremental response on repetitive nerve stimulation is absent [20]. A definite diagnosis of CIM requires the following criteria fulfilled: a. The critically ill patient develop limb weakness or difficulty weaning after non-neuromuscular causes such as heart and lung disease have been excluded; b. CMAP amplitudes are less than 80% of the lower limit of normal in two or more nerves without conduction block; c. SNAP amplitudes are more than 80% of the lower limit of normal; d. Needle EMG shows short duration, low-amplitude motor unit potentials with early or normal full recruitment, with or without fibrillation potentials in conscious and collaborative patients, or increased CMAP duration or reduced muscle membrane excitability on direct muscle stimulation in non-collaborative patients; e. A decremental response on repetitive nerve stimulation is absent; f. Muscle histopathological findings of primary myopathy [20]. A diagnostic flowchart on CIP/CIM has been proposed by Latronico and Bolton [20]. EMG in CIP/CIM diagnosis may sometimes be limited in ICU settings due to patients incorporation and electrical interference of other devices. Maximum inspiratory pressure, which is a global measure of inspiratory muscle strength, may serve as a surrogate parameter for the assessment of CIP/CIM [21].

GBS is a rare heterogeneous entity of autoimmune disorders (1.2 to 1.6 per 100,000 per year) occurring in previously healthy persons [22]. Patients usually had an infection 4–8 weeks prior to the onset, generally a flu-like episode or gastroenteritis, whose symptoms (fever, dyarrhea) have subsided by the time the neurological signs (pain, paraesthesias, numbness, and weakness in the limbs) become evident. Facial muscles are frequently involved. The axonal variants of GBS, i.e. acute motor axonal neuropathy and acute motor-sensory axonal neuropathy [23], may be more difficult to distinguish from CIP due to the similar clinical manifestations and EMG signs. Criteria of differentiation are specified in Table 1[24,25]. Although the clinical manifestations and EMG would not give supportive discrimination between the CIP/CIM and GBS, CSF analysis might be of help. A clinical hallmark of GBS is albumino-cytologic dissociation in CSF. The CSF protein concentrations of GBS patients are usually normal during the first week after onset of GBS, and are elevated in more than 90% of the patients at the end of the second week [26]. Because this patient developed symmetric flaccid weakness rapidly, we considered diagnosis of GBS initially. However, CIP/CIM may develop quite rapidly in some patients [27-29]. This case underscores the importance of differentiating CIP/CIM from GBS in case of such patients who develop weakness rapidly after the treatment.

**Table 1:** Differentiation between CIP and GBS

	**CIP**	**GBS**
Prodromal conditions	Sepsis, multiple organ failure, etc.	Gastrointestinal or respiratory infection
Clinical presentation	Onset of the disorder usually after ICU admission;	Onset of the disorder usually before ICU admission;
	Often be characterized by fairly symmetric limb muscle weakness sparing cranial nerves;	Infections precede the onset of progressive weakness and sensory disturbances;
	Sensory deficits less prominent	Frequent cranial nerve involvement
CSF	Usually normal	Albumino-cytologic dissociation
Electrophysiology	Axonal motor & sensory polyneuropathy	1. Demyelinating polyneuropathy or unresponsive nerves, abundant spontaneous activity
		2. Axonal motor & sensory polyneuropathy
MRI	No significant findings	Occasional enhancement of spinal nerve roots
Biopsy	Primarily axonal degeneration of distal peripheral nerves without inflammation	Primarily demyelinating process with inflammation, or motor/sensory axonal degeneration, or motor axonal degeneration only
Treatment	No specific therapy, usually anti-septic treatment	Plasmapheresis, intravenous immune globulin
Outcome	Recovery may be spontaneous and of variable timing; 50% of patients with full recovery	Usually >75% complete recovery

CIP: critical illness polyneuropathy; GBS: Guillain-Barré syndrome; ICU: intensive care unit; CSF: cerebrospinal fluid; MRI: magnetic resonance imaging.

**Figure 2:**
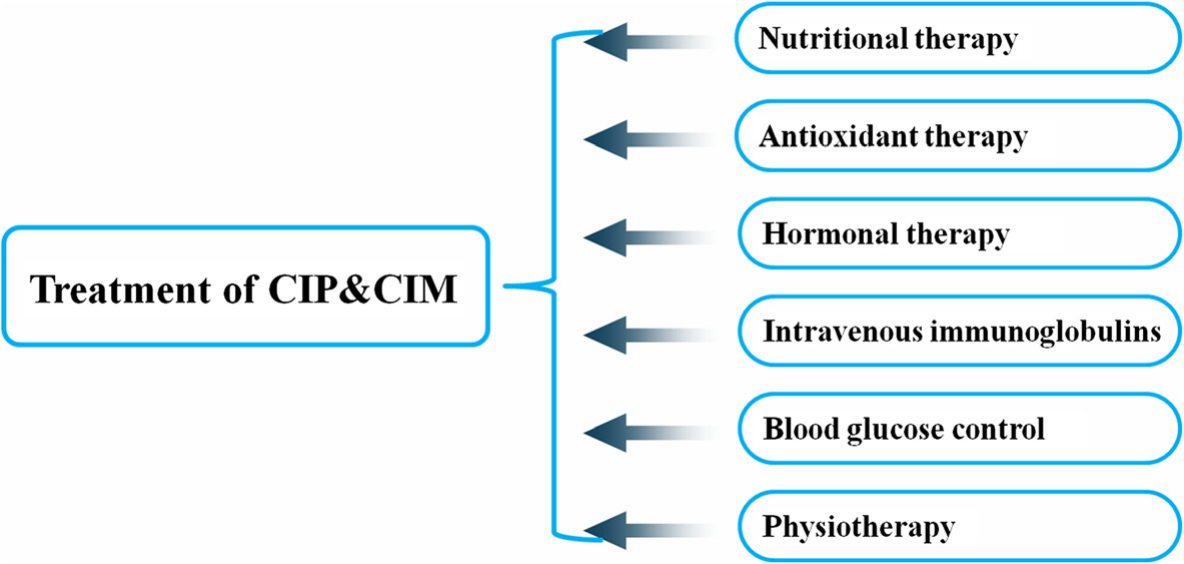
**Therapeutic strategies for CIP and CIM.** No specific therapy has been proved to be beneficial to manage CIP or CIM. Supportive measures including nutritional interventions, anti-oxidant therapies, hormone replacement, and immunoglobulins have been proposed. Intensive insulin therapy remarkably improves blood glucose control, and independently reduces the incidence of CIP/CIM. Early rehabilitation combining mobilization with physiotherapy is also advisable.

Supportive measures including nutritional interventions, supplement and anti-oxidant therapy, and the application of testosterone derivates, growth hormones and immunoglobulins were proposed to manage the muscle weakness in critically ill patients (Figure 2) [30]. Neuromuscular blocking agents (NMBAs) and corticosteroids should be used at minimal doses for as short a period as possible [20]. Insulin can alleviate the leakage of capillaries and enhanced permeability, inhibit the passage of neurotoxic factors into the endoneurium and impair the mitochondrial dysfunction from an increased generation/deficient scavenging of ROS. Intensive insulin therapy remarkably improves blood glucose control, and independently reduces the incidence of CIP/CIM (Figure 2) [31]. However, data from some other studies challenged the intensive insulin therapy, indicating that intensive glucose control may increase mortality among adults [32]. Recent studies have suggested that electrical stimulation (EMS) can reduce loss of muscle mass and exert an acute beneficial effect on microcirculation, thereby reducing the incidence of CIP/CIM and favorably affecting muscle strength [33]. EMS is also safe, tolerable and can be easily applied even in patients not able to cooperate. In addition to dealing with known risk factors and providing supportive measures, early rehabilitation is also important for the prevention and treatment of CIP/CIM. Early rehabilitation combining mobilization with physiotherapy is advisable to functional independence of the patients and the duration of ventilation and hospitalization is shortened [34,35]. Rehabilitation should be initiated as early as possible once the diagnosis is established [36].

## Conclusions

In summary, we reported a case of CIP and CIM as a severe complication of PNL. This case expands our understanding of the complications of PNL and underscores the importance of differentiating CIP/CIM from GBS in case of such patients developing weakness rapidly after the procedure. Diagnosis should be achieved at an early stage and treatment be initiated immediately. Both anti-septic prophylaxis and control of hyperglycemia might be effective for the prevention of CIP or CIM. Avoiding or limiting the use of corticosteroids and NMBAs, as well as EMS and early rehabilitation is also suggested.

## Consent

Written informed consent was obtained from the patient for publication of this Case report and any accompanying images. A copy of the written consent is available for review by the Series Editor of this journal.

## Abbreviations

ARDS: Acute respiratory distress syndrome;AST: Aspartate transaminase;IP: Critical illness polyneuropathy;CIM: Critical illness myopathy;CK: Creatine kinase;CMAP: Compound muscle action potential;CSF: Cerebrospinal fluid;DMS: Direct muscle stimulation;ECG: Electrocardiography;EMG: Electromyography;GBS: Guillain-Barré syndrome;ICU: Intensive care unit;IVU: Intravenous urography;PNL: Percutaneous nephrostolithotomy;MOF: Multiple organ failure;SNAP: Sensory nerve action potential;UPJ: Ureteropelvic junction;WBC: White blood cell

## Competing interests

The authors declare that they have no competing interests.

## Authors’ contributions

HL and LMW collected the history of the case and together with HY, RZ and HYL, drafted the manuscript. XBK critically reviewed the manuscript. HLZ finalized the manuscript. All authors read and approved the final manuscript.

## Pre-publication history

The pre-publication history for this paper can be accessed here:

http://www.biomedcentral.com/1471-2369/14/36/prepub
